# Hematopoietic Cell Transplant and Use of Massage for Improved Symptom Management: Results from a Pilot Randomized Control Trial

**DOI:** 10.1155/2012/450150

**Published:** 2012-02-09

**Authors:** Wolf E. Mehling, E. Anne Lown, Christopher C. Dvorak, Morton J. Cowan, Biljana N. Horn, Elizabeth A. Dunn, Michael Acree, Donald I. Abrams, Frederick M. Hecht

**Affiliations:** ^1^Osher Center for Integrative Medicine, University of California, San Francisco, CA 94115, USA; ^2^Department of Family and Community Medicine, University of California, San Francisco, CA 94115, USA; ^3^Alcohol Research Group, Public Health Institute, Emeryville, CA 94608, USA; ^4^Blood and Marrow Transplant Division, Department of Pediatrics, University of California, San Francisco, CA 94143, USA; ^5^Department of Medicine, Hematology and Oncology, University of California, San Francisco, CA 94110, USA; ^6^Department of Medicine, University of California, San Francisco, CA 94115, USA

## Abstract

*Background*. Pediatric hematopoietic cell transplant (HCT) is a lifesaving treatment that often results in physical and psychological discomfort. An acupressure-massage intervention may improve symptom management in this setting. *Methods*. This randomized controlled pilot trial compared a combined massage-acupressure intervention to usual care. Children were offered three practitioner-provided sessions per week throughout hospitalization. Parents were trained to provide additional acupressure as needed. Symptoms were assessed using nurses' reports and two questionnaires, the behavioral affective and somatic experiences scale and the Peds quality of life cancer module. *Results*. We enrolled 23 children, ages 5 to 18. Children receiving the intervention reported fewer days of mucositis (Hedges' g effect size ES = 0.63), lower overall symptom burden (ES = 0.26), feeling less tired and run-down (ES = 0.86), having fewer moderate/severe symptoms of pain, nausea, and fatigue (ES = 0.62), and less pain (ES = 0.42). The intervention group showed trends toward increasing contentness/serenity (ES = +0.50) and decreasing depression (ES = −0.45), but not decreased anxiety (ES = +0.42). Differences were not statistically significant. *Discussion*. Feasibility of studying massage-acupressure was established in children undergoing HCT. Larger studies are needed to test the efficacy of such interventions in reducing HCT-associated symptoms in children.

## 1. Introduction

 Hematopoietic cell transplant (HCT) can be a lifesaving treatment for cancer and other disorders. Despite advances in supportive care children suffer considerable physical and psychological discomfort during their hospitalization for HCT. Nonpharmacological means of symptom management are attractive adjuncts to care given their generally lower risk for additional side effects. However, there are limited data on such interventions in this patient population. 

Massage, borrowed from both Western and Eastern traditions, is a noninvasive modality to reduce symptom burden including pain, nausea, and anxiety. Massage studies have suggested benefits for oncology patients, [1–12] pediatric patients [13, 14] and for HCT recipients, adult [15] as well as pediatric [16], but the results from smaller studies of pediatric HCT recipients were not confirmed in a larger study [17]. Large observational studies of massage in adult cancer patients suggest benefits in the short and long term [1] but these findings are limited by the lack of a randomized control group.

Massage methods commonly used in cancer care include Western (Swedish) and Eastern (acupressure) massage styles [18, 19]. Both styles are increasingly being combined [20] but this combination has not been previously studied.

 Acupressure massage, the manual stimulation of specific acupuncture points commonly used in traditional Chinese medicine, can reduce chemotherapy-associated nausea [8, 15, 21–24], vomiting [25], anxiety, and fatigue [15, 26], and its use by massage practitioners has been increased in Europe [19, 27, 28] and the US [29]. A Cochrane review concluded that acupressure may be a low-cost, convenient, easily administered intervention for chemotherapy patients to reduce acute nausea [21]. Acupressure massage can be successfully taught to both patients and caregivers [30] and shows benefits for postoperative nausea in children in a meta-analysis [31]. Western massage is effective in reducing anxiety and pain [32, 33] mostly in the short term [34, 35], and cumulative or long-term effects have been reported [36].

Caregivers of family members with cancer experience high levels of distress [16, 37, 38], particularly mothers [39–42]. Frequently, parents of children with cancer experience symptoms of posttraumatic stress disorder (PTSD) [43, 44] with related health problems [45]. During the HCT process parents report feeling helpless in the face of their child's pain and suffering [43, 44]. This helplessness is a key risk factor for the development of PTSD [46]. Parental distress increases the risk for posttraumatic stress symptoms [47] and other adjustment problems in the child [48–50].

If parents are taught to perform massage on their children, both patient and caregiver may benefit. Parents who provide massage for sick children report reduced distress [16, 51, 52]. Parents reported improved self-efficacy in managing their child's symptoms, decreased anxiety, and decreased stress when they massaged their sick children [53–55].

 The aims of the present study were (a) to determine feasibility of a practitioner-provided combined massage and acupressure intervention for children undergoing HCT with parent training for additional parent-provided massages and (b) to collect preliminary data on the efficacy of this intervention for decreasing treatment-related symptoms such as nausea, vomiting, and pain. A secondary aim explored whether training parent caregivers to provide massage decreased parents' perceived stress and psychological distress and improved their sense of self-efficacy.

## 2. Methods

We conducted a randomized, nonblinded pilot study of a massage/acupressure intervention compared to usual care for pediatric patients undergoing HCT at an academic medical center. Imbalanced randomization (2 : 1) was used, stratified by allogeneic versus autologous transplantation, with more persons randomized to the massage/acupressure intervention to allow greater assessment of the feasibility of the intervention arm in this setting. Participants and study staff were aware of the group assignment. The study was approved by the university's Human Subjects Review Committees, and informed consent was obtained from participant's parents, and assent from participants over age 12. The study was registered with clinical trials.gov NCT00843180.

### 2.1. Patients

At the preadmission intake visit for HCT treatment, twenty-five consecutive children aged 5 to 18 years and their parents were invited to participate in the massage study. Twenty-three patients consented (92% response rate). Study personnel used sealed opaque envelopes that contained the computer-generated intervention or usual care group assignment. The envelopes were opened on the first day of hospital admission after baseline assessment was completed.

### 2.2. Intervention

Two experienced (>10 years) professional massage practitioners provided 20–30 minutes of semistandardized and manualized combined Swedish and acupressure massage three times a week in the patient's room over the entire duration of the hospital stay. Practitioners utilized: (a) a semistandardized Swedish-style massage for feet, legs and arms, and, when feasible, for back and shoulder girdle; (b) acupressure massage used specific pain, nausea, and calming acupressure points selected based on prior research [56–58] and consultation with acupuncture experts: Pericardium 6 (wrist) and Stomach 36 (below the knee) for nausea; Triple Warmer 5 (wrist) for stress; Large Intestine 4 (hand) and Liver 3 (foot) for pain; Kidney 6 and Bladder 62 (ankle) for nausea/stress; Spleen 6 (above the ankle) for stress. The practitioners instructed parents on how to use acupressure on their child demonstrating point location on the child and on the parent (for purposes of learning the point location) and using an instruction sheet with pictures, location descriptions, and indications for 9 selected points. Parents were encouraged to perform additional acupressure on their child for improved and timely symptom management.

Control subjects received usual care: state-of-the-art medical treatment for HCT with pharmaceutical symptom management for pain and nausea but no massages or acupressure. They were offered a single 20-minute massage each for parent and child in the days before discharge and separate $25 gift cards for child and parent after completion of all questionnaires.

### 2.3. Measures

Two types of data were collected in both groups: nurse's daily clinical records and questionnaire data. Questionnaires were administered to both parents and children to assess the child's physical and psychological symptoms. Parents also answered questions about their own wellbeing. Nurses' clinical data included information for each day on: nausea, number of vomiting episodes, pain, and mucositis. Most were assessed at multiple points during the day. Data were collected on days of hospital stay and days to absolute white cell and neutrophil count greater than 500 cells/mm³ for 3 consecutive days.

Questionnaires were administered to children and parents by the research assistant at baseline and then every two weeks. One week after discharge from inpatient care, the parent was interviewed over the phone. Questionnaires for children age 5–7 used simplified response options and face symbols. Data in the current report are based on child report.

Physical symptoms and psychological states were assessed using the BASES and PedsQL. The *behavioral affective and somatic experiences scale (BASES)* is a 22-item instrument developed at St. Jude Children's Research Hospital specifically for the assessment of child distress during hospitalization for HCT. Independent subscales measure somatic distress, compliance, mood/behavior (anxious, depressive), interactions, and activity. Both child and parent report BASES were used [59, 60]. To decrease patient burden, we reduced the number of items to 18 by dropping two less relevant subscales (compliance, interactions). The *PedsQL Cancer Module *[61, 62] is a 28-item modular quality of life measure for children with cancer. The cancer module is appropriate for an inpatient setting and includes versions for age groups: 5–7, 8–12, and 13–18 years old and for parents. It includes eight independent subscales; we used those corresponding to problems with pain (2 items), nausea (5), procedural anxiety (3), and worry (1), thereby reducing the number of items to 12. Positive affect was measured using four items selected from the *differential emotions scale (DES)* [63].

Parents were given an additional questionnaire administered at baseline and in the week following discharge from the hospital. The questionnaire included the *CES-D* [64] measure of depression [65] and the *parent's self-efficacy scale *[53]* (PSES)*, which assessed self-efficacy of managing their child's symptoms. Posttraumatic stress symptoms were assessed using the *PTSD symptom scale *[66]* (PSS) *[67].

### 2.4. Outcomes

Primary outcomes included symptoms of fatigue, nausea, vomiting, and pain. Secondary outcomes included mucositis and worry/anxiety. Several composite variables were derived from the nurses' daily clinical notes and from parent and child questionnaire data. The primary composite variable was derived classifying moderate-severe symptoms using the three key symptoms of pain, nausea, and fatigue using child self-report data from the BASES sub-scales. A second summary measure was derived from nurses' data and included number of days of any pain >3 (on a scale of 1–10), any nausea, any vomiting, and any mucositis.

### 2.5. Analyses

Analysis of the data was performed using SAS version 9.2. We compared baseline data between groups to assess the success of randomization by demographic and cancer characteristics and psychological status using chi square statistics. Physical symptom scores measured by questionnaire and nurses' clinical records were compared in intention-to-treat analyses using *t*-tests. Nurses' multiple daily electronic medical records were used for the time period of 7 days before to 21 days after-transplant and children's self-report data at baseline and one week after transplant. As the intervention in this pilot study was provided during the children's entire hospital stay, outcome measures were collected during the entire hospital stay as well in order to assess the feasibility of data collection in this setting. However, for the purpose of providing efficacy data on effect sizes needed for sample-size calculations for a larger study, we limited the analysis of children *self-report* data to a narrower period, as patients began getting discharged before the self-report measures at 21 days were obtained. Standardized Hedges' g effect sizes were calculated taking uneven group sizes into account. Psychological status was assessed using change scores between baseline and one week following the transplant to control for baseline psychological state.

## 3. Results

**Figure 1:**
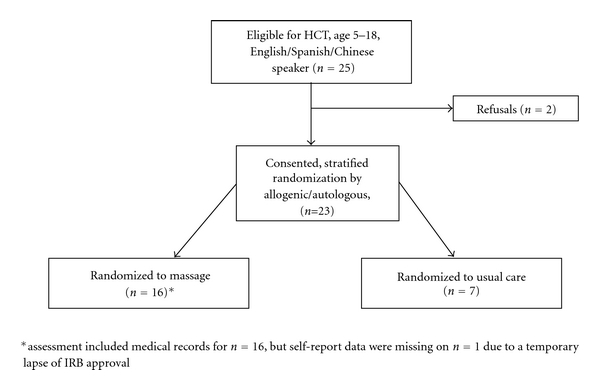
Participant flow chart.

Twenty-five children aged 5 to 18 years old and their rooming-in parents were admitted for HCT at the university children's hospital between November 2008 and December 2009 and invited to participate; 23 consented and enrolled. Patient characteristics are summarized in Table 1. Sixteen children were randomly assigned to the massage group and 7 to usual care (Figure 1).

During a median hospitalization of 41 days, children in the intervention group received a median of 8.5 massages averaging 1.6 massages per week. Fourteen of the 16 parents (87%) reported performing massages on their children. Children and parents completed all requested surveys and a postdischarge telephone survey. No adverse side effects for the intervention were reported.

Five children were discharged from the hospital before the 3-week posttransplant survey; thus the focus of the data analysis is on the nurse's daily notes and the one-week posttransplant self-report data. This small feasibility study was not expected to provide sufficient power to show statistically significant differences between groups; thus we report standardized effect sizes that allow for sample-size calculations for future studies.

Results for key symptoms as reported in the nurse's daily notes are summarized in Table 2. None of the symptoms showed statistically significant improvements in the massage/acupressure group. However, we did find some large-to-moderate effect sizes (ES) in favor of the intervention in several important outcomes. Based on nurse's data, children in the intervention group had fewer days of mucositis (ES = 0.63) and lower overall symptom burden (ES = 0.26). Data from the child's self-report also did not show statistically significant benefits for the massage group, but showed a trend toward improved fatigue, ES = 0.86, *P* = 0.08. In addition the intervention group reported fewer moderate/severe symptoms in a summary measure of fatigue, pain, and nausea (ES = 0.62) and decreased pain (ES = 0.42). There were no statistically significant between-group differences in duration of hospital stay or days to engraftment.

Findings related to psychological outcome measures (Tables 3(a) and 3(b)) are reported using change scores. The intervention group showed more self-reported beneficial changes for depression (ES = −0.45) and contentness/serenity (ES = +0.50). Sleep was reported to have changed in a negative direction in the intervention group (ES = −0.96); likely a spurious finding, as a worsening of sleep as a result of our intervention does not make sense and was not confirmed in qualitative interviews. Parental outcomes of self-efficacy, perceived stress, posttraumatic stress symptoms, and mood showed no differences between groups at the time of hospital discharge.

## 4. Discussion

Our results demonstrated the feasibility of providing and studying a combined Swedish massage and acupressure intervention in a pediatric HCT unit. While the sample size was small, the data suggested some efficacy of the massage/acupressure intervention, particularly related to a reduction in days with mucositis, improvements in fatigue, and reduced pain and loss of appetite. Use of daily nurse's clinical data combined with biweekly self-report data from both children and parents provided multiple perspectives on the clinical efficacy of the intervention for our key outcomes. While the effect sizes we observed are encouraging, the results must be interpreted cautiously given the small sample size and lack of statistically significant differences between groups. A larger study would be needed to determine whether the effect sizes suggested in this pilot study can be confirmed with statistically significant results. Based on our results for a key study outcome—the summary score of the three key symptoms of pain, nausea, and fatigue (ES = 0.62), the study would have required at least 64 participants in each group to show a statistically significant difference.

The feasibility of the study was further supported by enthusiastic qualitative data obtained from parent interviews and nurses' reports. These findings are reported separately in a detailed qualitative report (manuscript submitted) [68, 69].

There are a number of unique aspects of the present pilot study compared to other studies in the field of massage and pediatric oncology. The present pilot tested an integration of Eastern and Western massage styles, as is increasingly practiced in the United States. The potential benefits include the relaxing aspects of Swedish massage [1, 33] combined with the potential efficacy of acupoint therapy for pain, nausea, and other symptom relief [22]. To our knowledge, there are no comparable studies that have tested an integration of Eastern and Western massage. Involvement of parents in providing additional nonprofessional massages is another innovative feature of our intervention with the added benefits of increasing the massage dose, supporting timely symptom management and enabling parents to help their children directly.

A previous single-site pilot study performed by Phipps and colleagues [16] demonstrated promising results in improved symptom management, but these results were not confirmed in a larger multisite study [17]. The intervention used in this larger study, a combination of a laugh cart, a guided relaxation and Western massage was substantially different from the present study, with most overlap in the shared aim of reducing child discomfort by nonpharmacological means. The present study may have benefited from the additional use of acupressure, which may account for the moderate-to-high effect sizes for some symptoms compared to this prior study.

Another small feasibility study in 17 children with cancer who were undergoing chemotherapy used a crossover design in which 4 weekly massage sessions alternated with 4 weekly quiet-time control session. This study found that massage was more effective than quiet time at reducing heart rate and anxiety in children less than 14 years but did not show improvements in pain, nausea, or fatigue [70]. The authors concluded that massage in children with cancer is feasible and appears to decrease anxiety. The present study also showed feasibility, but otherwise found different results with moderate ES for a decrease in pain and fatigue and less improved anxiety in the intervention group compared to controls. The addition of acupressure in the present study may have improved symptom management over the previous study, but sample sizes are small in both studies.

Finally, a randomized feasibility study of acupressure in preventing chemotherapy-associated nausea by Jones and colleagues was conducted among 21 pediatric oncology patients, ages 5 to 19 years, using wrist bands compared to placebo bands [71]. Acupressure applied using wrist bands was feasible and well tolerated but there were not statistically significant results compared to placebo*, potentially due to small sample size. *While Jones's study used a bead to apply pressure on one acupoint, it differed from the present study, in which acupressure was provided by experienced practitioners who used multiple points.

The finding that the intervention may have reduced days with mucositis and reduced tiredness, although at first surprising, may in fact reflect a potential mechanism of action that has been suggested by results from prior acupoint studies, namely, a reduction in proinflammatory cytokines such as TNF-*α*, IL-1, and IL-6 [72]. These proinflammatory cytokines are increased during chemotherapy, probably due to high levels of apoptosis (programmed cell death). Some of these proinflammatory cytokines, in turn, are hypothesized to be important factors in chemotherapy-related fatigue [73] and mucositis [74]. This hypothetical mechanism may deserve further investigation in a larger trial with the addition of biological samples.

The present study focused on longer-term changes in symptoms (assessed every other week from self-report or daily by the nurses) rather than short-term changes (minutes or hours) after the intervention. This design allowed us to assess only the more enduring effects of massage, but not the immediate effects. Future studies might benefit from both short- and longer-term assessments. Short-term benefits from massage have been reported in other studies [75] and appear to be more consistent than longer-term effects.

Limitations of this feasibility study include the small sample size and the limited number of time points for self-report assessments. In addition, the dose of the massage intervention averaged 1.8 massages per week, while the target dose was 3 per week. This difference occurred in part because of scheduling difficulties related to periods of time with severe symptoms, unscheduled naps, and high health care demands. Participants often preferred massage sessions in the evening when medical procedures were over, but massage providers had limited evening availability. Finite resources made it difficult to ensure wider availability of the massage provider. Based on our results, we believe that an increased dose of massage would be facilitated by having a provider regularly available during the late afternoon and evening for several hours per day (rather than individually scheduled visits).

Major strengths of the study include the feasibility and acceptability of a massage/acupressure study on a busy pediatric stem cell transplant unit, indications of efficacy of the intervention, the lack of side effects, and the enthusiastic support for the intervention by the involved pediatricians, nurses, and parents [68, 69]. 

This study provided new data on the efficacy of combined Swedish massage and acupressure for improved symptom management in children undergoing hematopoietic cell transplants. Findings from this and larger future studies have the potential to influence clinical practice related to stem cell transplant-associated symptoms in children by introducing massage and acupressure, an ancient healing modality, into a “high-tech” pediatric hospital setting. Massage and acupressure for symptom management are attractive, given their potential to treat multiple symptoms with few or no side effects. Future studies should enroll sufficient numbers to better test the efficacy of combined Swedish massage and acupressure in symptom management of pediatric hematopoietic stem cell transplant.

## Figures and Tables

**Table 1:** Patient characteristics.

	Intervention	Control
*N *(intention to treat):	16	7
Demographics		
Age (mean) (range 5–18)	11.3	13.9
Sex		
Female	7	3
Male	9	4
Ethnicity		
White	9	2
Asian	3	5
Hispanic	3	0
Other	1	0
Diagnoses		
Congenital or acquired Bone marrow failure	5	0
Hematologic malignancy	6	5
Congenital immune deficiency	3	1
Solid tumor	2	0
Hemoglobinopathy	0	1
Transplant type		
Autologous Allogeneic	13 3	6 1

**Table:** (a) Symptoms from the nurses records over 29 days (7 days before to 21 days after transplant). Intervention (I; *N* = 16) versus control (C; *N* = 7).

Symptom (scale range)	Arm	Mean ± SD	ES	*p*°

Days of pain > 3 (on 0–10 scale)	I C	7.8 ± 4.9 8.0 ± 5.5	0.04	0.93
Days of vomiting	I C	5.7 ± 4.6 5.2 ± 5.0	0.11	0.82
Days of mucositis	I C	9.9 ± 7.1 14.7 ± 8.9	0.63	0.18
Immune recovery (Days until 3 days of WBC > 500)	I C	17.1 ± 5.1 17.9 ± 10.1	0.12	0.85
Days with high symptom burden (pain > 3, nausea, mucositis, and vomiting)*¹*	I C	1.8 ± 0.5 2.0 ± 0.6	0.26	0.57

**Table:** (b) Symptoms from child self-report: at baseline and 1 week after transplant. Intervention (I; *N* = 15) versus control (C; *N* = 7).

Symptom (scale range)	Arm	Mean ± SD		ES	*p*°
		Before	After		

Nausea and vomiting*¹* (0–4)	I C	0.3 ± 0.7 1.1 ± 1.3	2.3 ± 1.5 2.3 ± 1.5	+0.01	0.98
Loss of appetite*¹* (0–4)	I C	1.3 ± 0.7 2.0 ± 1.7	2.8 ± 1.5 3.3 ± 1.0	+0.36	0.44
Feeling tired/run down*¹* (0–4)	I C	1.5 ± 1.3 1.0 ± 0.8	1.9 ± 1.3 3.0 ± 1.0	+0.86	0.08
Pain*¹* (0–4)	I C	0.8 ± 1.0 1.3 ± 1.5	1.2 ± 1.2 1.6 ± 0.8	+0.42	0.37
Summary score of 3 moderate/severe symptoms of fatigue, pain, and nausea¹** (0–3)	I C	0.3 ± 0.4 0.3 ± 0.8	1.1 + 1.1 1.7 + 0.8	+0.62	0.23

*¹*Higher score: worse symptoms.

*ES: standardized effect sizes (“+” ES is advantage for intervention; “−” ES is advantage for control).

°*t*-test.

SD: standard deviation.

**Sum of moderate or severe (“quite a bit/very much” versus “none/a little/somewhat”) symptoms of fatigue, nausea, and pain, 1 week self-report (range: 0–3 symptoms).

All measures were from the BASES questionnaire except pain was measured using the Peds quality of life scale.

**Table:** (a) Changes in negative affect from baseline to 1 week after transplant. Mean “+” change indicates worsening; mean “−” change indicates improvement; ES “−” indicates benefit for intervention compared with control; ES “+” vice versa.

Symptom change (scale range)	Arm	Means ±SD			ES	*p*°
		Before	After	Change		

Depression*¹* (0–4) by child report	I C	0.7 ± 0.9 0.7 ± 0.8	0.9 ± 1.1 1.4 ± 1.0	+0.2 ± 1.1 +0.7 ± 1.1	−0.45	0.33
Anxiety*¹* (0–4) by child report	I C	0.9 ± 1.0 1.4 ± 1.1	0.6 ± 1.0 0.7 ± 0.8	−0.3 ± 0.9 −0.7 ± 1.0	+0.42	0.37
Worry*¹* (0–4) by child report	I C	1.4 ± 1.1 1.7 ± 1.4	1.4 ± 1.2 1.3 ± 1.3	−0.0 ± 1.2 −0.4 ± 1.2	+0.32	0.50
Complaining/demanding*¹* (0–4) by parent report	I C	2.4 ± 1.5 2.0 ± 1.5	2.1 ± 1.0 2.1 ± 1.2	−0.3 ± 1.7 +0.1 ± 0.7	−0.45	0.21

**Table:** (b) Changes in positive affect and sleep quality from baseline to 1 week after transplant. Mean “+” change indicates improving; mean “−” change indicates worsening; ES “+” indicates benefit for intervention compared with control; ES “−” vice versa.

Symptom change (scale range)	Arm	Means ±SD			ES	*p*°
		Before	After	Change		

Contentness/serenity² (0–4) by child report	I C	2.3 ± 1.4 2.1 ± 1.3	2.2 ± 1.5 1.3 ± 0.8	−0.1 ± 1.6 −0.9 ± 1.1	+0.50	0.29
Overall mood² (0–4) by child report	I C	2.8 ± 0.9 2.9 ± 0.7	2.3 ± 0.9 2.3 ± 1.0	−0.4 ± 1.2 −0.6 ± 0.8	+0.20	0.72
Sleep³ (0–4) by child report	I C	2.7 ± 1.1 2.3 ± 1.0	2.4 ± 1.3 2.7 ± 0.8	−0.3 ± 0.8 +0.4 ± 0.5	−0.96	0.05

*¹* Higher score: worse negative affect.

² Higher score: higher positive affect.

³ Higher score: better sleep.

* ES: standardized effect sizes.

°*t*-test.

I: intervention, C: control.

SD: Standard Deviation.

(Sample *N* for intervention (I) versus control (C); intention to treat: 15 versus 7).
